# Research trends related to emergence agitation in the post-anaesthesia care unit from 2001 to 2023: A bibliometric analysis

**DOI:** 10.1515/med-2024-1021

**Published:** 2024-09-02

**Authors:** Lulu Cao, Yunhong Ren, Fang Wen, Juan Du, Mei He, Huaping Huang

**Affiliations:** Department of Endoscopic Center, Mianyang Central Hospital, School of Medicine, University of Electronic Science and Technology of China, Mianyang, Sichuan, 621900, China; Department of Anesthesiology, Mianyang Central Hospital, School of Medicine, University of Electronic Science and Technology of China, Mianyang, Sichuan, 621900, China; Nursing Department, Mianyang Central Hospital, School of Medicine, University of Electronic Science and Technology of China, Mianyang, Sichuan, 621900, China

**Keywords:** emergence agitation, VOSviewer, bibliometric analysis, Web of Science Core Collection, global trends

## Abstract

**Background:**

Emergence agitation (EA) is a behavioural disturbance encountered during the recovery phase of patients following general anaesthesia. It is characterised by restlessness, involuntary limb movements, and drainage tube withdrawal and may significantly harm patients and medical staff. The mechanism of EA has not been fully understood and is still a challenging subject for researchers.

**Methods:**

We extracted relevant publications published between 1 January 2001 and 31 December 2023 on the Web of Science Core Collection platform. VOSviewer software was utilised to analyse the retrieved literature and predict the development trends and hotspots in the field.

**Results:**

The results show that the number of publications grew annually, with China contributing the most, followed by the United States and South Korea. The co-occurrence of keywords “children,” “propofol,” “risk factors” are current research hotspots. Owing to its self-limiting and short-duration characteristics, EA lacks standardised clinical time guidelines and objective assessment tools, which may be the focus of future research in this field.

**Conclusions:**

Understanding the research hotspots and the latest progress in this field, this study will help to continuously improve the clinical understanding and management of EA, and help to timely identify environmental risk factors for EA in clinical practice.

## 1 Introduction

Emergence agitation (EA) is an acute mental disorder that occurs during the period of recovery from general anaesthesia. It is characterised by the co-occurrence of excitement, agitation, and disorientation, accompanied by involuntary limb movement, crying or groaning, and other inappropriate behaviours [1]. At this time, patients are in the recovery stage of consciousness, muscle strength, and internal environment, and they lack self-awareness and protective reflexes. This can easily lead to self-removal of oxygen masks, tracheal catheters, infusion lines, catheters, and gastric tubes, and other adverse consequences, resulting in hypoxia, surgical incision dehiscence and bleeding, and other complications, affecting patients’ surgical recovery time and even be life-threatening [2]. Thus, the effective prevention and timely treatment of restlessness during recovery in patients under general anaesthesia has become a key quality indicator for resuscitation. Understanding the hotspots and new progress in this field is helpful in identifying and dealing with the potential risk factors of EA and helping patients under general anaesthesia recover better. Therefore, this study used the Web of Science Core Collection (WoSCC) platform to comprehensively analyse the research status of EA.

The WoSCC is recognised as the most reliable and influential database platform for obtaining global-level academic information [3]. It can conduct multi-angle and visual panoramic analyses of search results and summarise the development trends of related research fields, subject distribution, and relevant journal contributions. Through a multi-angle and comprehensive in-depth analysis, the developmental trend and current situation of the discipline can be revealed at the macro-level [4].

Bibliometrics is a discipline that studies the distribution, structure, and relationship of literature and information using quantitative analysis methods such as mathematics and statistics. It then discusses and evaluates the trends and rules of research development [5]. The results of bibliometric analysis have been applied in multiple medical fields, such as oncology, orthopaedics, nursing, and basic medicine [6–9]. However, bibliometric studies on EA are lacking. Therefore, this study aimed to conduct a systematic analysis of EA research to identify research trends and hotspots in this field and highlight new directions for future research.

## 2 Methods

### 2.1 Study search and selection

**Figure 1:**
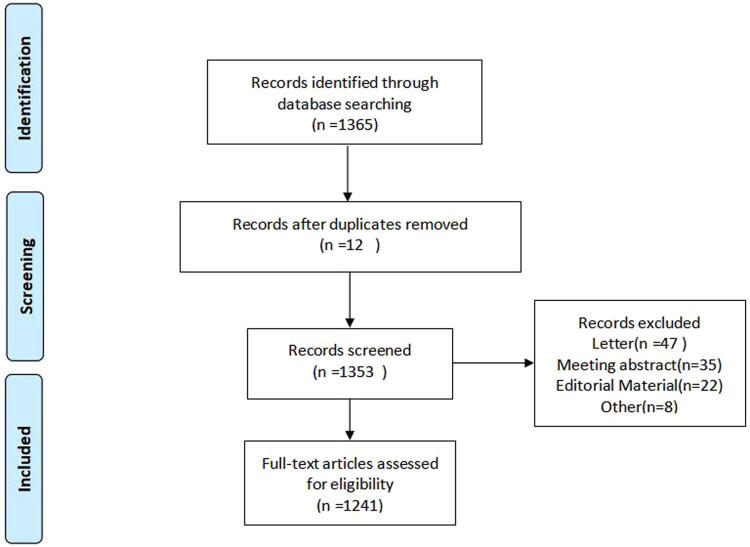
Literature screening flow chart.

All studies related to EA from 1 January 2001 to 31 December 2023 were extracted from the WoSCC (including Science Citation Index Expanded, Social Sciences Citation Index, and Conference Proceedings Citation Index-Science). The constructed retrieval formula is as follows: TS = (agitation OR delirium) AND TS = (emergence) AND DOP = (2001-01-01/2023-12-31) AND LA = (English). “Tab-delimited files” for document export and ‘full records and cited references’ for record content were selected. A total of 1,241 records conforming to the standards were imported into VOSviewer, along with the publication information, literature sources, cooperation information, keywords, and other literature content. A flowchart of the literature selection process is shown in Figure 1.

### 2.2 Bibliometric analysis

VOSviewer version 1.6.19 (Centre for Science and Technology Studies, Leiden University; based on JAVA) is a bibliometric analysis software that creates web-based maps and provides visualisations (network, coverage, and density visualisations) of maps and exploration maps to help researchers evaluate literature research directions and hotspots [10]. VOSviewer draws a clustering graph based on the literature co-citation principle, where each colour represents a subtopic, and each node represents a literature. The larger the node, the more cited the literature; the closer the node examples, the closer the relationship between the literature; and the thicker the line, the more co-cited the two literatures. Keyword co-occurrence clustering maps are used to show the degree of correlation between research topics. The author collaboration network map can identify representative authors and research teams in a research field.

## 3 Results

### 3.1 Global publication trend

**Figure 2:**
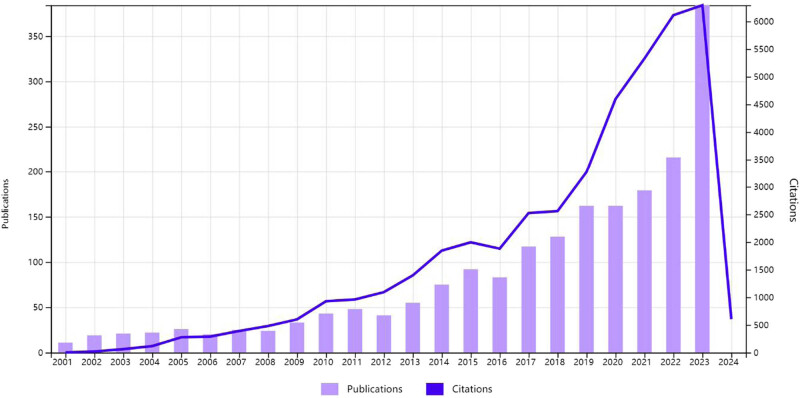
Times cited and publications over time.

As shown in Figure 2, the number of publications in the EA field is generally increasing, and researchers’ interest in the field continues to grow. From seven articles in 2001 to 138 in 2023, the number of publications increased by approximately 20 times in 23 years. During 2019–2022, the growth rate of the cited frequency was higher than that of the number of publications. The 1,241 literature that met the requirements were cited 29,308 times, with an average of 23.62 times; the total *h*-index was 77.

### 3.2 Analysis of distribution of co-authors and institutions

**Figure 3:**
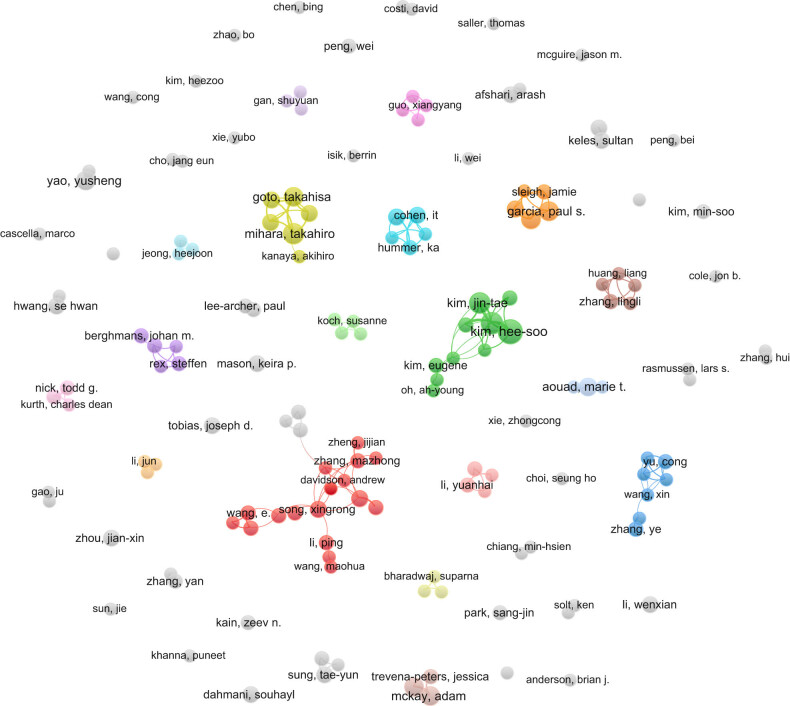
Analysis of co-author distribution.

**Figure 4:**
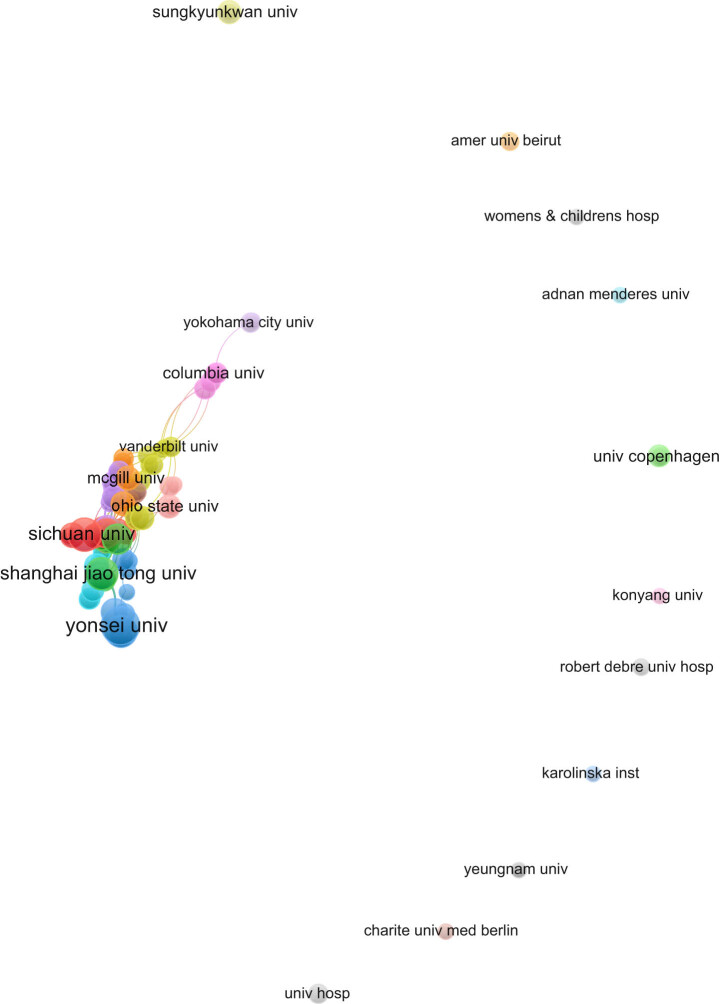
Analysis of the organisation’s distribution of co-authors.

Between 2001 and 2023, approximately 1,532 institutions from 66 different countries/regions produced literature on EA. The analysis was of co-authorship; the minimum required number of documents by an author was three, and the minimum number of publications by an institution was five. Out of the 5,900 authors analysed, 154 met the threshold, and of the 1,532 institutions, 96 met the threshold. Hee-soo Kim of the Department of Anesthesia and Pain Medicine at the Seoul National University Hospital published the most papers (11 articles), followed by Ji-Hyun Lee from the same hospital (9 articles). The authors with the most publications are shown in Table 1. The top ten universities in terms of the number of published papers are shown in Table 2, among which the top three are Yonsei University (31 articles), Sichuan University (23 articles), and Seoul National University (21 articles). Figure 3 shows that there is less cooperation among scholars; cooperation among authors is limited to internal members, and no real academic community has been formed. According to Figure 4, there is a certain degree of cooperation among the major issuing institutions but without a central trend.

**Table 1:** Top ten authors in terms of publication

Author	Documents	Citations	Total link strength
Kim, Hee-soo	11	117	28
Lee, Ji-hyun	9	85	27
Kim, Jin-tae	8	76	26
Mihara, Takahiro	8	68	22
Garcia, Paul S.	7	65	9
Goto, Takahisa	7	68	21
Kreuzer, Matthias	7	58	14
Mckay, Adam	7	77	10
Ponsford, Jennie	7	155	10
Yao, Yusheng	7	121	3

**Table 2:** Top ten organisations in terms of publication

Organisations	Documents	Citations	Total link strength
Yonsei Univ	31	735	9
Sichuan Univ	23	160	4
Seoul Natl Univ	21	371	8
Shanghai Jiao Tong Univ	21	107	13
Harvard Med Sch	18	445	17
Capital Med Univ	17	104	5
Korea Univ	15	220	8
Zhejiang Univ	14	165	6
Chongqing Med Univ	13	88	6
Sun Yat Sen Univ	13	105	3

### 3.3 Analysis of country distribution

**Figure 5:**
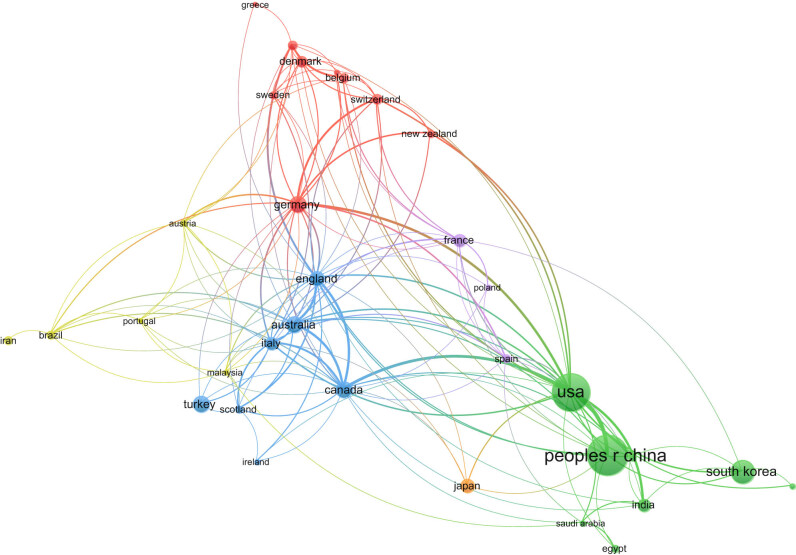
Analysis of country distribution.

To some extent, the number of EA publications in different countries reflects the research situation in this field. When the minimum number of publications by a country was set to a default of five, 34 countries met the threshold. In Figure 5, each node represents a country, and the size of the node represents the number of published papers in this country. China had the largest number of nodes, with 340 published papers, accounting for 27.4% of the total number. This was followed by the US with 303 publications and South Korea with 117. The number of publications in other countries is listed in Table 3. Figure 5 shows that the United States and China cooperated closely, followed by the United States and Canada. There were 151 connections between 66 countries, with a total link strength of 338, among which connections between European and American countries were the most frequent.

**Table 3:** Top ten countries in terms of publication

Country	Documents	Citations	Total link strength	Percentage
Peoples R China	340	2,989	33	27.4
USA	303	11,847	97	21.32
South Korea	117	2,053	10	9.43
Turkey	59	1,347	6	5.36
Canada	56	2,086	56	4.75
Germany	56	1,673	55	4.75
Australia	55	1,327	47	4.43
England	47	1,212	61	3.79
Japan	41	794	6	3.3
France	36	1,092	19	2.9

### 3.4 Analysis of journals

Over the past 23 years, 429 journals have published articles on EA. Table 4 lists the ten journals with the highest number of published articles, along with their impact factor (IF), Quartile in category (2022). The top five journals are *Pediatric Anesthesia*, *Anesthesia and Analgesia, BMC Anesthesiology, European Journal of Anesthesiology*, and *British Journal of Anaesthesia.* Among the top ten journals in terms of the number of publications, the highest IF was for the *British Journal of Anaesthesia*, followed by the *Journal of Clinical Anesthesia*, *Anesthesia and Analgesia*. The IF scores of the above three journals were all greater than 5, and they were all rated Q1 in the Journal Citation Reports classification for 2022.

**Table 4:** Top ten journals in terms of publication

Journal title	Documents	Citations	IF (2022)	Quartile in category (2022)
Pediatric Anesthesia	105	3,160	1.7	Q4
Anesthesia and Analgesia	44	2,970	5.9	Q1
BMC Anesthesiology	43	399	2.2	Q3
European Journal of Anaesthesiology	33	594	3.6	Q2
British Journal of Anaesthesia	32	2,202	9.8	Q1
Acta Anaesthesiologica Scandinavica	31	878	2.1	Q4
Journal of Clinical Anesthesia	27	418	6.7	Q1
Journal of Perianesthesia Nursing	25	177	1.7	Q3
Medicine	24	209	1.6	Q3
Journal of Anesthesia	22	562	2.8	Q3

### 3.5 Analysis of keywords

**Figure 6:**
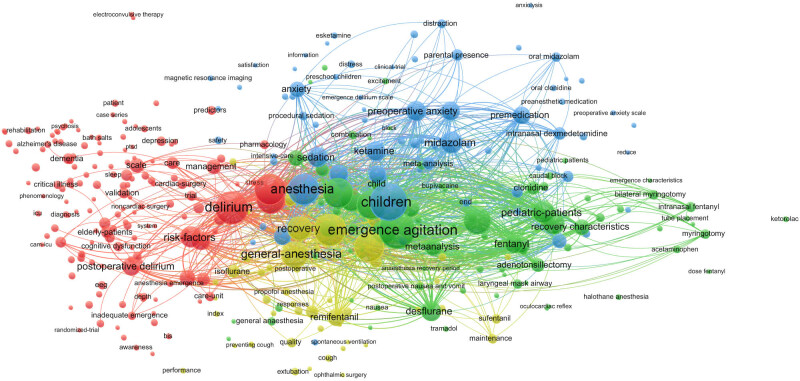
Frequency analysis of keyword occurrence.

**Figure 7:**
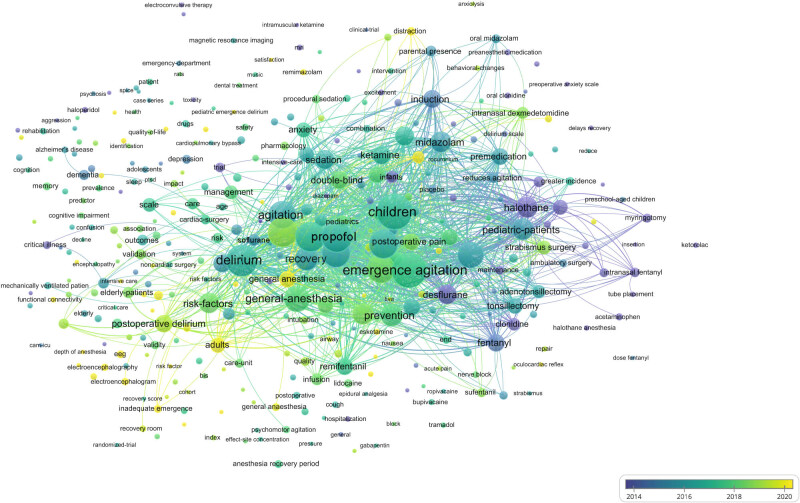
Analysis of changes in keywords by year.

**Figure 8:**
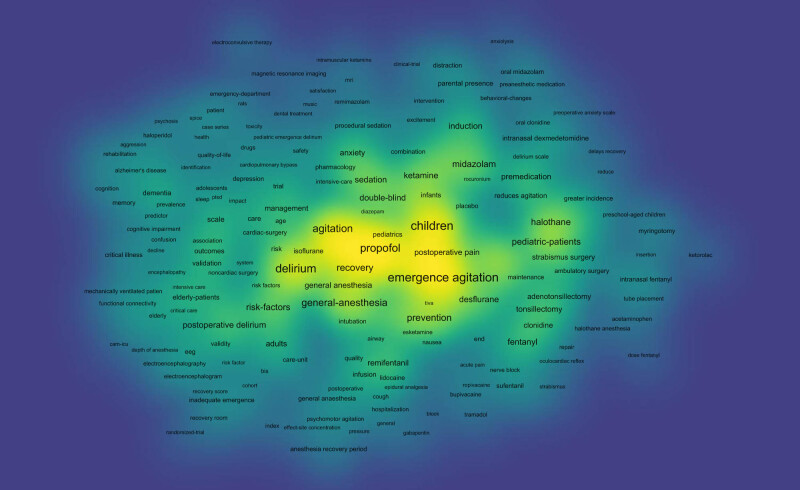
Analysis of keyword hotspot.

In bibliometrics, the analysis of keywords is critical because it serves as the core of the article and is more representative in the presentation of domain analysis due to its more condensed information [11]. VOSviewer was used to extract and create statistics on the keywords in the included literature. The minimum number of keyword occurrences was set as five by default. A total of 3,931 keywords were found, and 361 met the threshold; the top 30 high-frequency keywords are listed in Table 5. The size of each node represents the weight of the keyword, and the distance between the two nodes represents the connection between the two keywords; the closer the connection, the shorter the distance. The colour of the node represents the respective clustering. The cluster size was adjusted to 25, and four clusters were generated, as shown in Figure 6. Keyword overlay visualisation takes the score values for the average year of the keyword and colour-maps them to examine emerging themes within the research domain and the temporal evolution of the research direction. The closer the node is to yellow, the more novel the theme is, as shown in Figure 7. Density visualisation was used to examine the density of studies within the domain. The larger (smaller) the number of neighbouring items and the higher (lower) their weights, the closer the colour of the point is to yellow (blue), as shown in Figure 8.

**Table 5:** Frequency of keywords

Rank	Keywords	Frequency of occurrence	Total linkage strength
1	Children	369	3,264
2	Emergence agitation	358	3,184
3	Delirium	284	2,470
4	Propofol	280	2,599
5	Anaesthesia	261	2,190
6	Emergence delirium	256	2,068
7	Surgery	235	2,130
8	Sevoflurane	234	2,259
9	Dexmedetomidine	223	2,085
10	Agitation	215	1,894
11	Sevoflurane anaesthesia	197	1,903
12	General-anaesthesia	187	1,698
13	Recovery	157	1,358
14	Prevention	141	1,363
15	Paediatric-patients	135	1,403
16	Halothane	128	1,283
17	Midazolam	127	1,282
18	Pain	127	1,101
19	Risk factors	120	984
20	Sedation	106	893
21	Desflurane	105	1,052
22	Preoperative anxiety	102	940
23	Ketamine	95	838
24	Double-blind	92	776
25	Emergence	91	680
26	Induction	89	824
27	Fentanyl	87	887
28	Postoperative delirium	84	661
29	Paediatric anaesthesia	82	707
30	Premedication	82	861

## 4 Discussion

This study applied the bibliometric analysis method of VOSviewer to analyse the annual publication amount, journal distribution, publication institution and country distribution, high-frequency keyword analysis, keyword contribution clustering, timeline, and density visualisation analysis and comprehensively sorted out the EA-related research results. This will help readers understand the research status, trends, and hotspots in this field.

The number of publications in the field of EA has increased annually over the past 5 years, indicating that the development of EA-related research is rapid and that the field of EA has received increasing attention from researchers. The bibliometric analysis of journals not only reflects the academic influence of journals in the research field but also helps researchers select their contributions. Furthermore, the IF is a general international index for the overall evaluation of journals. It is not only an indicator of the usefulness and degree of the journals, but it is also a crucial indicator of the academic level of journals and the quality of papers. Therefore, our bibliometric analysis and examination of the IF highlight the validity of the current research on EA. Notably, *Pediatric Anesthesia*, with relatively low influence, was the journal with the largest number of publications. This suggests that research on children’s EA requires more attention.

EA is inappropriate behaviour in the anaesthesia recovery period, manifested as excitement, agitation, and disorientation; inappropriate behaviour, such as involuntary limb movements, irrational speech, crying or moaning, delusional thinking, and so on; serious cases can cause harm to the patient and related nursing staff; and patients can remove the mask and pull out the catheter, resulting in hypoxia, wound opening, bleeding, and other serious consequences. Although the mechanisms through which EA occurs are unclear [12,13], several factors are associated with postoperative agitation. Recent studies suggest that postoperative pain and the pharmacokinetics and pharmacodynamics of anaesthetics are related to EA. Lim et al. [14] found that sevoflurane may induce hyperexcitatory behaviour after anaesthesia by enhancing the depolarisation or excitation effect of some 1-aminobutyric acid neurons in the neocortex of the brain. Based on animal model studies, neuroinflammation and neurotransmitters may be one of the pathophysiological mechanisms of EA, which requires more evidence from human studies [15–17]. Furthermore, previous studies and clinical reviews found that the occurrence of EA may be related to multiple risk factors, such as age, sex, type of surgery, method of anaesthesia, use of anaesthetic drugs, preoperative mental state, and preoperative drug use [2,18,19]. The prevention, timely identification, and control of EA are key, and the combination of prevention and control can minimise the incidence of EA.

As shown in Figure 7, the risk factors leading to EA have been the focus of research in recent years. Among adult patients, the incidence of EA is higher in men than in women, and EA is more common in children than in adults [2,18]. Different types of surgery, due to differences in surgical sites, often need intraoperative indwelling drainage tubes or indwelling gauze haemostasis. Therefore, patients often feel pain, which influences the occurrence of EA. Voepel-Lewis et al. [20], in a prospective cohort study of 521 children aged 3–7 years who underwent general anaesthesia for outpatient surgery, found that ophthalmological and otorhinolaryngological procedures were associated with EA. Among these, otorhinolaryngological procedures have been shown to be an independent risk factor for EA. Figure 6 shows that the keywords “adenotonsillectomy,” “adenoidectomy,” “tonsillectomy,” and “surgery” have high frequencies of occurrence, indicating that they are the current research hotspots. In addition, different surgical methods of the same type of surgery may affect the occurrence of EA [21]. There are studies [19,22] retrospectively compared the risk of EA in patients undergoing open gastrectomy (OG) with those undergoing laparoscopic gastrectomy and found that the higher incidence of EA in patients with OG may be related to diaphragmatic motor dysfunction caused by postoperative incision.

Ramroop et al. [23] showed that compared to elective surgery, the higher incidence of EA after emergency surgery is related to inadequate preoperative preparation and uncorrected internal environment disorders in patients undergoing emergency surgery. The use of narcotic drugs is an important risk factor for EA. Keywords such as “dexmedetomidine,” “sevoflurane anaesthesia,” “halothane,” “desflurane,” “sufentanil,” “propofol,” “sevoflurane,” “intraoperative dexmedetomidine,” and “fentanyl” have the highest frequency, indicating that these are the current research hotspots. Inhaled anaesthetics are widely used, especially for paediatric patients, because of their rapid-acting and counteracting advantages. However, the use of volatile anaesthetics increases the risk of EA compared to total intravenous anaesthesia (TIVA) [24,25]. The solubility of different drugs in the blood can affect the incidence of EA. A randomised, double-blind study of 144 adult patients undergoing orthodontic surgery noted a lower incidence of EA with desflurane than with sevoflurane anaesthesia [26]. Some studies have shown that the incidence of EA in children under desflurane anaesthesia is higher than that of those under sevoflurane anaesthesia, and children under desflurane anaesthesia can wake up faster, which may be caused by different subjects, different types of surgery, and different rating scales used. Hence, the conclusions are inconsistent, and relevant research conclusions need to be further verified [27,28]. Inadequate analgesia is an essential factor in EA, and the effective use of opioids suggests that pain is associated with postoperative agitation [29]. A prospective, randomised, double-blind, multicentre study of the incidence of EA after adenoidectomy and tonsillectomy in children under general anaesthesia found that nalbuphine reduced the incidence of EA by reducing postoperative pain [12]. Although postoperative pain is a key trigger of EA, painless surgery can also cause it. Dalens et al. [30] demonstrated that children under sevoflurane anaesthesia may develop EA during an MRI examination; therefore, the mechanism of pain and EA requires further examination.

The occurrence of EA is related to multiple factors, and the timely identification of risk factors and timely diagnosis will help to better prevent and deal with this phenomenon. Currently, EA does not have globally uniform diagnostic criteria, and the scoring scales used by children and adults are different. Paediatric anaesthesiologists generally agree that the psychologically-tested Pediatric Anesthesia Emergence Delirium (PAED) scale developed in 2004 is the most effective and reliable one [31,32], and it can be used for children over 2 years of age [33]. The scale includes five items on consciousness and cognition, psychomotor behaviour, and emotional change, and the scores for each item are added together to obtain the PAED score. The higher the score, the heavier the EA degree [33]. However, the shortcomings of the PAED score are that it is too subjective, there are differences between evaluators, and some behaviours in the evaluation indicators overlap with other postoperative negative behaviours. There is controversy about the threshold value of the PAED score criteria [32–34]. The main tools used to evaluate EA in adults include the Richmond agitation sedation scale and sedation-agitation scale, both of which are subjective rating scales to evaluate the depth of sedation in ICU patients [35,36]. The reliability of the scale is high, but the reliability of the diagnosis of EA in convalescent patients under general anaesthesia is unknown. The subjective sedation assessment scale commonly used in clinical practice is simple and fast but has some problems; therefore, it is necessary to introduce objective assessment tools, such as the bispectral index and quantitative electroencephalogram, to assist in the diagnosis of EA and improve its diagnostic accuracy [18,37,38]. Therefore, the timely detection of patients with EA and effective interventions are needed.

Understanding the pathogenesis and mechanisms of EA and its active prevention and treatment are of great significance for patient safety and surgical success. Pharmacological and non-pharmacological methods are the directions of our efforts, and studies have demonstrated that TIVA is more appropriate than inhalational anaesthesia for high-risk EA [39]. However, further rigorous prospective research comparing the effects of balanced anaesthesia and TIVA on EA is required [40]. The use of propofol throughout anaesthesia may mitigate EA in paediatric patients undergoing general anaesthesia; however, further investigation is warranted to determine its efficacy in adult populations [18,41].

“Fentanyl” was a keyword with a high frequency of occurrence. A meta-analysis of randomised controlled trials found that prophylactic fentanyl administration reduced the incidence of EA after sevoflurane anaesthesia [42]. Furthermore, dexmedetomidine is a highly selective α_2_-adrenoreceptor agonist with sympathetic, inhibitory, analgesic, and sedative effects [43]. A randomised controlled study involving 100 adult patients undergoing nasal surgery demonstrated that the intraoperative infusion of dexmedetomidine maintained stable haemodynamics and reduced the incidence of EA without delaying extubation [44]. Non-pharmacological methods, such as informing patients of predictable postoperative pain or discomfort, removing the indwelling catheter as soon as possible, and the presence of parents during anaesthesia induction and resuscitation, can reduce the incidence of EA [18,45,46].

EA is a common pathological state after general anaesthesia. The pathogenesis and diagnostic criteria of EA in adults have not yet been clarified. Whether the changes in brain electrical activity and brain metabolism are potential mechanisms needs clinical verification and further research. At present, there are many subjective evaluation scales for sedation of patients, which are simple and quick to use, but they are not widely used in EA patients, and lack of reliability and validity verification of clinical data. In the future, the subjective scale assessment combined with objective sedation monitoring methods can be widely studied in clinical practice to guide the treatment of EA.

## 5 Conclusion

This study used bibliometric methods to analyse the characteristics and evolution of international research in the past 22 years and summarises the global research trends and hotspots of EA, and provide important references for researchers in this field to understand the advantageous clusters and new research directions in this field. China, with the largest number of articles (340), made the largest contribution. The most authoritative findings are available in the *British Journal of Anaesthesia* and the *Journal of Clinical Anesthesia.* Hee-soo Kim and Ji-hyun Lee published the most papers in this field, with a focus on EA in children, halothane, adenotonsillectomy for timely identification of risk factors, and management of EA high-risk patients (application of drug and non-drug strategy). The diagnostic criteria for EA in adults are not yet clear and more reliable, and effective objective monitoring indicators need to be explored to predict and prevent EA. The primary limitation of this study is that only the literature of the WoSCC database was retrieved to ensure the standardisation of data, and this data lacked certain integrity. Second, with English literature as the data source and non-English literature excluded, the research results are not comprehensive.
